# Addressing the Rejection of a Rebuttal Letter: Author’s Experience and Thoughts

**DOI:** 10.31662/jmaj.2024-0102

**Published:** 2024-09-06

**Authors:** Hideharu Hagiya

**Affiliations:** 1 Department of Infectious Diseases, Okayama University Hospital, Okayama, Japan

**Keywords:** baroxavil, evidence-based medicine, influenza, risk of bias, systematic review and meta-analysis

Writing rebuttal letters to published papers is crucial for upholding the validity of science, particularly in medicine. No matter how meticulously constructed, clinical studies potentially carry a risk of bias, which threatens the quality and certainty of evidence. Herein, I share my bitter experience of submitting a rebuttal letter to a medical journal, intending to provoke discussions on this intractable issue.

Recently, I read an article in the *Journal of Infection and Chemotherapy* entitled “Comparison of clinical efficacy and safety of baloxavir marboxil versus oseltamivir as the treatment for influenza virus infections: A systematic review and meta-analysis” ^(1)^. The respective authors compared the clinical efficacy and safety between baroxavil (BXM) and oseltamivir, with all-cause mortality as the primary outcome and the hospitalization duration and incidence of adverse events as the secondary. Their methodology seems well-organized, and they concluded that safer and more effective results were observed among patients treated with BXM than those with oseltamivir. Accordingly, the authors provided a favorable recommendation for prescribing BXM for the initial treatment of patients with influenza. However, because of the limited number of enrolled studies, their results need careful evaluation.

Despite an exhaustive search, only two each of randomized controlled trials (RCTs) and retrospective studies were identified for outpatients and inpatients, respectively. Compared with that of patients treated with oseltamivir, an analysis of inpatients treated with BXM revealed a lower mortality tendency (3.3% vs. 6.0%: *p* = 0.06) and a significantly shorter hospitalization duration (mean: 12.5 days vs. 25.8 days: *p* = 0.01). Mortality was analyzed using two retrospective analyses, whereas hospitalization duration data were based on the results of a single study. Although this study was designed as a systematic review and meta-analysis, its primary and secondary outcomes were based on only two retrospective studies. Concerning the Preferred Reporting Items for Systematic Reviews and Meta-Analyses (PRISMA) guidelines for reporting systematic reviews and meta-analyses ^(2)^, there is a discrepancy in their evaluation concerning “Risk of bias in studies” (Item #18) and “Results of individual studies” (Item #19). Moreover, the mean hospitalization duration for patients treated with BXM and oseltamivir were approximately 2 and 4 weeks. Influenza is an acute respiratory infection that typically resolves within several days to 1 week at most. The excessively long hospitalization durations in both groups suggest that most of the enrolled patients had potentially confounding clinical conditions―besides influenza―requiring prolonged hospitalization. Thus, their evaluation of the certainty for each measured outcome seemed inadequate in contravention of the requirements outlined in “Certainty of evidence” (Item #22) of the PRISMA guidelines ^(2)^.

Consequently, it might be an overestimation to conclude that the BXM treatment can effectively reduce hospitalization durations among inpatients. The excessive use of BXM may lead to the emergence of BXM-resistant influenza strains ^(3), (4)^. Too much emphasis on the clinical superiority of BXM may be misleading based on the results of this study and should be avoided.

I have documented and submitted these medical concerns to the “Letter to the Editor” section of the *Journal of Infection and Chemotherapy*. However, my letter was rejected without a convincing explanation. The International Committee of Medical Journal Editors delineates the preferable state of medical debate in their recommendations (Correspondence section) as follows: “*Medical journals should provide readers with a mechanism for submitting comments, questions, or criticisms about published articles, usually but not necessarily always through a correspondence section or online forum. The authors of articles discussed in correspondence or an online forum have a responsibility to respond to substantial criticisms of their work using those same mechanisms and should be asked by editors to respond*” ^(5)^. In addition, it denotes that “*responsible debate, critique and disagreement are important features of science, and journal editors should encourage such discourse ideally within their own journals about the material they have published*.” I believe that the critiques and opinions stated herein are concise and hold significant value, warranting open discussion to foster scientific discourse.

Notably, the problem is the absence of an established place or platform to assert scientific opinions when a rebuttal letter is rejected by the respective journals. What solutions are currently available and should be sought in such a situation? We should now discuss this issue, lingering from the past to the present.

## Article Information

### Conflicts of Interest

None

### Author Contributions

HH drafted and revised the manuscript.
